# Free-choice Mongolian herbal-mineral supplementation improves serum mineral, antioxidant, endocrine, and metabolic indices in rams with pica

**DOI:** 10.3389/fvets.2026.1946016

**Published:** 2026-10-14

**Authors:** Da Man, Lexiang Chen, Xiaofang Ao, Wenguang Zhang, Risu Na

**Affiliations:** 1 College of Animal Science, Inner Mongolia Agricultural University, Hohhot, China; 2 State Key Laboratory of Animal Nutrition and Feeding, College of Animal Science and Technology, China Agricultural University, Beijing, China

**Keywords:** antioxidant defense, herbal–mineral supplementation, pica, porphyrin metabolism, rams, serum metabolomics

## Abstract

**Introduction:**

Pica is a common abnormal feeding behavior in sheep and is often associated with mineral imbalance, oxidative stress, and metabolic dysfunction; however, the responses of affected sheep to herbal–mineral supplementation remain poorly characterized. This study evaluated the effects of a free-choice Mongolian herbal–mineral supplement (MHMS) on serum mineral concentrations, antioxidant capacity, biochemical and endocrine indices, and serum metabolomic profiles in rams with pica.

**Methods:**

Twenty East Friesian rams with pica (1.5 ± 0.2 years of age; 42.6 ± 3.8 kg body weight) were randomly allocated to a control group receiving a basal diet (CON, *n* = 10) or a group receiving the same basal diet with free-choice access to MHMS (MHMS, *n* = 10). The intervention lasted for 60 days following a 14-day adaptation period, and serum samples were collected at the end of the trial.

**Results:**

Compared with the CON group, the MHMS group had higher serum Cu, Zn, Se, triiodothyronine, thyroxine, insulin-like growth factor 1, and growth hormone concentrations and greater total antioxidant capacity, but lower serum glucose and cortisol concentrations and aspartate aminotransferase activity (*p* < 0.05). Untargeted serum metabolomic analysis putatively annotated 1,062 metabolites. Using the criteria of variable importance in projection (VIP) > 1.0, *p* < 0.05, and fold change (FC) ≥ 1.5 or ≤ 0.67, 112 candidate differentially abundant metabolites were detected, including 51 with higher abundance and 61 with lower abundance in the MHMS group. Pathway analysis highlighted porphyrin metabolism, in which glycine abundance was higher and porphobilinogen abundance was lower in the MHMS group.

**Discussion:**

Overall, these findings suggest that free-choice MHMS supplementation may influence trace-element status and related antioxidant, endocrine, and metabolic responses in rams with pica.

## 1 Introduction

Pica is a common abnormal feeding behavior in sheep production systems, typically characterized by the persistent ingestion of non-feed substances such as wool, soil, and urine, or by licking walls, stones, and metal fences (1). Pica not only disrupts normal feeding activity but may also increase the risk of gastrointestinal injury, impaired nutrient absorption, parasitic infection, and reduced production performance (2, 3). Currently, the etiology of pica has not been fully elucidated. However, it is generally considered to be a multifactorial disorder involving nutritional imbalance, metabolic disturbance, environmental stress, and other factors (1, 4). Among these factors, mineral deficiency or imbalance has been widely recognized as a major cause of pica. Deficiencies in elements such as phosphorus, copper, iron, zinc, and selenium may disrupt antioxidant defense capacity and metabolic homeostasis, thereby promoting the development of pica (4–6).

Dietary mineral supplementation can partly correct mineral deficiencies and represents a nutritional strategy for preventing or alleviating pica. In a study on wool-eating behavior in lambs, dietary supplementation with FeSO₄, CuSO₄, and ZnSO₄ increased serum Fe, Zn, and Cu levels and resulted in complete recovery of the affected lambs after 8 weeks of treatment (7). The development of pica may also involve oxidative stress, endocrine dysregulation, or metabolic dysfunction; therefore, mineral supplementation alone may not be sufficient to fully correct abnormal feeding behavior (8, 9). Indeed, the efficacy of mineral supplementation depends not only on the amount supplied but also on factors such as the chemical form of the minerals, mineral antagonisms, the rumen environment, and gastrointestinal absorption mechanisms. Thus, mineral supplementation does not necessarily translate into complete and effective mineral utilization (10, 11).

Plant-derived feed additives are increasingly used in livestock nutrition because they contain diverse bioactive constituents, may act through multiple targets and have a low risk of residues. As an important component of the medicinal plant resources characteristic of the Mongolian Plateau, Mongolian herbs are rich in bioactive compounds such as flavonoids, polyphenols, terpenoids, polysaccharides, and alkaloids, and may exert potential antibacterial, antioxidant, anti-inflammatory, and immunomodulatory effects (12). In ruminants, plant-derived feed additives have been reported to affect rumen fermentation, antioxidant status, immune function, blood biochemical parameters, and productive performance (13, 14). Relevant studies have shown that Arula-7 powder, a traditional Mongolian compound herbal formula, reduced diarrhea incidence in calves infected with pathogenic *Escherichia coli* O1, improved weight gain and serum biochemical parameters, enhanced antioxidant capacity and immune function, and improved intestinal epithelial barrier function by regulating gut microbiota structure and increasing the expression of tight junction proteins, including zonula occludens-1 (ZO-1), Occludin, and Claudin-1 (15). Combined supplementation with a herbal mixture and organic zinc may help reduce nematode infection in lambs, exerting an indirect effect by enhancing host resistance to parasitic infection (16). Supplementation with a blend of oregano essential oils and cobalt lactate has been shown to improve growth performance, crude protein digestibility, carcass traits, plasma metabolic profiles, and immunoglobulin levels in lambs (17). In addition, essential oil blends may not only affect rumen fermentation but also promote the absorption of cations such as Na^+^, Ca^2+^, and NH₄^+^ across the ruminal epithelium through direct interactions with epithelial transport proteins, thereby contributing to the regulation of calcium homeostasis and nitrogen metabolism (18). Shariga-5, a traditional Mongolian multi-herb formula derived from local ethnoveterinary practice, is characterized by high levels of bioactive flavonoids and phenolic acids. Combining Shariga-5 with essential trace minerals was hypothesized to exert synergistic effects, potentially facilitating mineral utilization and physiological recovery in sheep with pica.

Existing studies on nutritional interventions for sheep with pica have mainly focused on the alleviation of clinical signs and changes in individual mineral levels, whereas it remains unclear whether a Mongolian herbal–mineral supplement can induce systemic physiological and metabolic responses. In particular, it remains unclear whether such supplements can simultaneously regulate serum biochemical status, antioxidant function, endocrine status, mineral homeostasis, and the metabolome, and few studies have reported whether the underlying mechanisms involve energy metabolism, amino acid and lipid metabolism, oxidative stress, or endocrine-related pathways. Therefore, the present study aimed to evaluate the effects of free-choice MHMS supplementation on serum trace element concentrations, biochemical parameters, antioxidant capacity, hormone levels, and untargeted metabolomic profiles in sheep with pica. We prespecified the hypothesis that MHMS supplementation would directly improve serum trace-element concentrations, enhance antioxidant defense, and alleviate endocrine stress. Concurrently, untargeted metabolomics was employed as an unbiased, exploratory tool to capture systemic metabolic alterations without restrictive *a priori* pathway assumptions, thereby providing mechanistic clues into the physiological recovery of sheep with pica.

## 2 Materials and methods

### 2.1 Composition and preparation of the Mongolian herbal–mineral supplement

The target mineral and vitamin concentrations and the inclusion levels of the herbal ingredients in the Mongolian herbal–mineral supplement (MHMS) are presented in Tables 1, 2, respectively. These formulation values represent design specifications rather than analytically determined concentrations. The formulation was developed at Inner Mongolia Agricultural University. The raw materials were processed and manufactured into 5-kg solid lick blocks by Inner Mongolia Bushi Biotechnology Co., Ltd. One MHMS lick block was placed in each pen assigned to the MHMS treatment and remained continuously available for free-choice consumption throughout the experimental period.

**Table 1:** Target concentrations of minerals and vitamins in the Mongolian herbal–mineral supplement.

Nutrient	Target concentration^1^
Ca (mg/kg)	50,000
P (mg/kg)	30,000
Cu (mg/kg)	30
Fe (mg/kg)	500
Mn (mg/kg)	300
Zn (mg/kg)	750
Se (mg/kg)	20
Co (mg/kg)	5
Vitamin D_3_ (IU/kg)	50,000
Vitamin E (IU/kg)	500

Ca, calcium; Co, cobalt; Cu, copper; Fe, iron; IU, international unit; Mn, manganese; P, phosphorus; Se, selenium; Zn, zinc.

^1^Formulation values for the Mongolian herbal–mineral supplement are design values.

**Table 2:** Composition of the Shariga-5 herbal formula used in the Mongolian herbal–mineral supplement.

Herbal ingredient (Mongolian name)	Scientific name	Plant part	Inclusion level^1^ (g/kg)
Shariga	*Curcuma longa* L.	Rhizome	1.20
Saolong-Jimusi	*Forsythia suspensa* (Thunb.) Vahl	Fruit	0.53
Gaoletu-Baori	*Syzygium aromaticum* (L.) Merr. & L.M. Perry	Flower bud	1.07
Arula	*Terminalia chebula* Retz.	Fruit	0.40
Zhurula	*Gardenia jasminoides* Ellis	Fruit	0.80

^1^Total herbal inclusion level = 4.0 g/kg supplement.

### 2.2 Animals, feeding, and experimental design

The experiment was conducted at a sheep farm in Hohhot, Inner Mongolia Autonomous Region, China. Before random allocation, candidate sheep were observed daily for seven consecutive days across two fixed 1-h sessions each day (09:00–10:00 and 15:00–16:00). Sheep exhibiting a cumulative frequency of 5–10 instances per observation day of one or more predefined non-nutritive oral behaviors—including geophagia, trichophagia, urine drinking, iron-fence licking or biting, or plastic chewing or ingestion—were confirmed as having pica and enrolled as experimental animals. Twenty male East Friesian sheep meeting these criteria (1.5 ± 0.2 years of age; 42.6 ± 3.8 kg body weight) were randomly allocated to one of two treatment groups, with 10 sheep per group. The sheep were housed in 20 pens, with one sheep per pen, resulting in 10 replicate pens per treatment group. Sheep in the control group (CON) received the basal diet alone, whereas those in the MHMS group received the same basal diet and had continuous free-choice access to Mongolian herbal–mineral supplement (MHMS) lick blocks. Free-choice access indicated that the lick block was continuously available ad libitum. Neither lick-block disappearance nor individual MHMS intake was recorded. The trial lasted 74 days, comprising a 14-day adaptation period followed by a 60-day experimental period. Sheep were fed the basal diet twice daily at 07:00 and 17:00 and had free access to water throughout the experiment (Figure 1). The composition of the basal diet is presented in Table 3.

**Figure 1:**
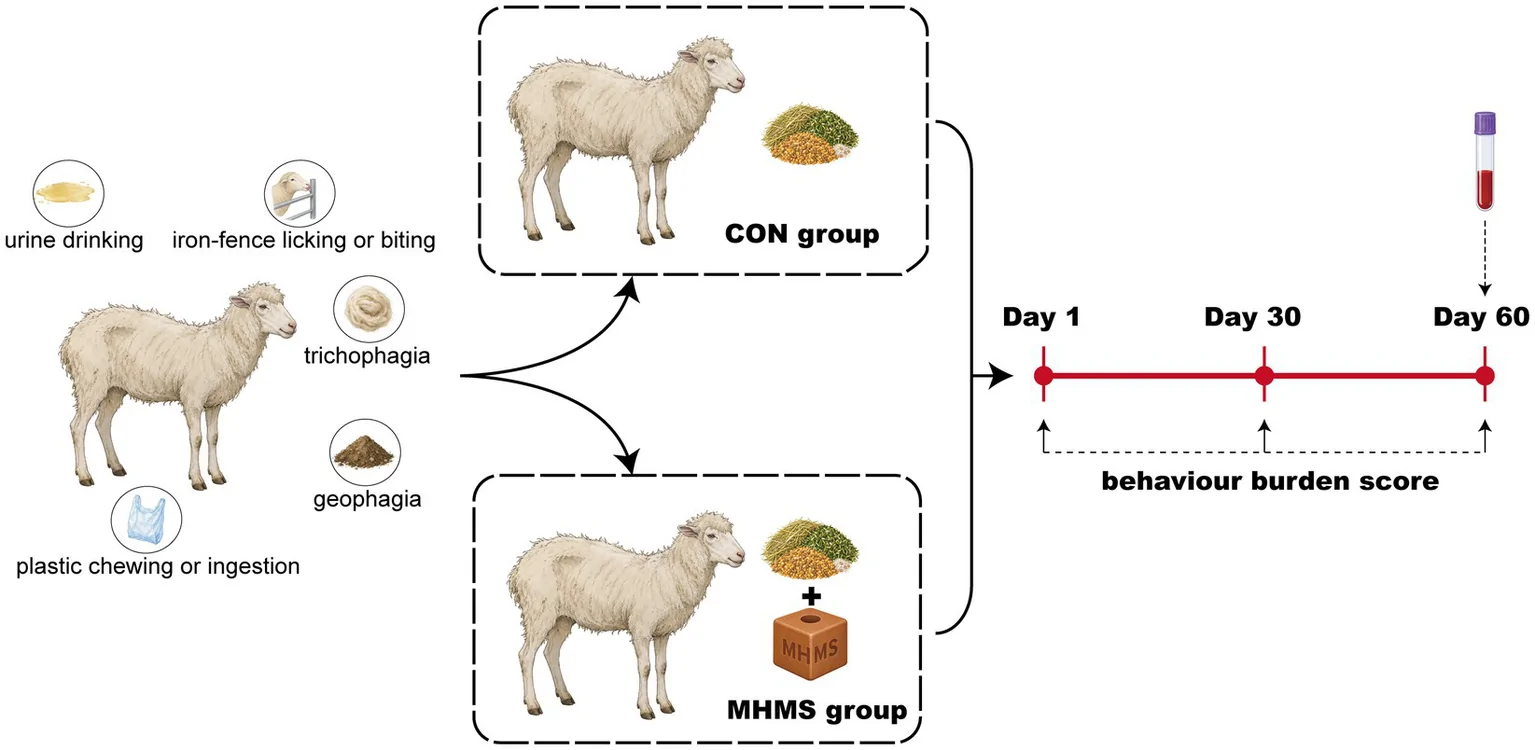
Experimental design and sampling workflow for the free-choice MHMS supplementation study in sheep with pica.

**Table 3:** Ingredient and nutrient composition of the basal diet (DM basis, % of DM).

Ingredient	Amount
Corn silage	31.25
Oat hay	27.86
Alfalfa hay	18.83
*Leymus chinensis* hay	21.46
Sodium bicarbonate	0.60
Nutrient composition (% of DM)^1^	
CP	11.60
EE	1.60
Ash	6.29
NDF	51.92
ADF	31.30
ME^2^, MJ/kg DM	8.41
Ca	0.73
P	0.41

DM, dry matter; CP, crude protein; EE, ether extract; NDF, neutral detergent fiber; ADF, acid detergent fiber; DE, digestible energy; ME, metabolizable energy; Ca, calcium; P, phosphorus.

^1^Nutrient composition values were analytically determined, except for metabolizable energy (ME).

^2^Metabolizable energy (ME) was estimated according to the Feeding Standard of Sheep of China (77). ME (MJ/kg DM) = 0.046 + 0.820 × DE (MJ/kg DM), DE (MJ/kg DM) = 17.211–0.135 × NDF (% DM).

### 2.3 Blood collection and serum preparation

On day 60 of the experimental period, before the morning feeding, 10 mL of blood was collected from each of the 20 sheep by jugular venipuncture into serum collection tubes without anticoagulant. The samples were allowed to clot at room temperature for 30 min and then centrifuged at 3,000 × g for 10 min at 4 °C. The resulting serum was divided into aliquots and stored at −80 °C until analysis. Serum samples from the same six randomly selected sheep in each treatment group were subsequently used for mineral, biochemical, antioxidant, hormonal, and metabolomic analyses.

### 2.4 Serum biochemical, antioxidant, hormonal, and mineral analysis

Serum glucose (GLU), total protein (TP), albumin (ALB), urea (UREA), total cholesterol (CHO), triglyceride (TG), high-density lipoprotein cholesterol (HDL-C), and low-density lipoprotein cholesterol (LDL-C) concentrations, together with alanine aminotransferase (ALT) and aspartate aminotransferase (AST) activities, were determined using an automated biochemical analyzer (BS-360E, Shenzhen Mindray Bio-Medical Electronics Co., Ltd., Shenzhen, China) and commercial reagent kits supplied by Dirui Industrial Co., Ltd. (Changchun, China). GLU was measured using the glucose oxidase method, TP using the biuret method, ALB using the bromocresol green method, and UREA using the urease–glutamate dehydrogenase method. CHO and TG were determined using the cholesterol oxidase–peroxidase aminophenazone (CHOD–PAP) and glycerol-3-phosphate oxidase–peroxidase aminophenazone (GPO–PAP) methods, respectively. HDL-C was measured using a direct catalase-clearance method, whereas LDL-C was measured using a direct surfactant-clearance method. ALT and AST activities were determined using alanine- and aspartate-substrate methods, respectively.

Serum antioxidant indices were determined using commercial colorimetric assay kits supplied by Jiangsu Aidisheng Biotechnology Co., Ltd. (Yancheng, China). Catalase (CAT) activity was measured using a residual-hydrogen peroxide colorimetric method, glutathione peroxidase (GSH-Px) activity using an organic peroxide substrate method, superoxide dismutase (SOD) activity was determined using the water-soluble tetrazolium salt-8 (WST-8) method, malondialdehyde (MDA) concentration using the thiobarbituric acid method, and total antioxidant capacity (T-AOC) using the ferric-reducing antioxidant power method. Absorbance was measured using a SpectraMax 190 absorbance microplate reader (Molecular Devices, San Jose, CA, USA).

Serum triiodothyronine (T3), thyroxine (T4), insulin-like growth factor 1 (IGF-1), growth hormone (GH), and cortisol (CORT) concentrations were measured using sheep-specific commercial ELISA kits supplied by Jiangsu Jingmei Biotechnology Co., Ltd. (Jiangsu, China). The catalog number, assay range, and limit of detection were JM-07869S2, 0.1–12 pmol/L, and <0.1 pmol/L for T3; JM-07865S2, 1.0–240 nmol/L, and <1.0 nmol/L for T4; JM-00419S2, 1–160 ng/mL, and <1.0 ng/mL for IGF-1; JM-00440S2, 0.1–16 μg/mL, and <0.1 μg/mL for GH; and JM-07861S2, 50–1,600 pg/mL, and <10.0 pg/mL for CORT, respectively. Absorbance was measured at 450 nm using an Infinite F50 microplate reader (Tecan, Männedorf, Switzerland), and hormone concentrations were calculated from the corresponding standard curves.

Serum calcium (Ca), inorganic phosphorus (P), magnesium (Mg), sodium (Na), potassium (K), iron (Fe), copper (Cu), zinc (Zn), manganese (Mn), and selenium (Se) concentrations were determined using colorimetric assays performed on an automated biochemical analyzer (BS-420, Shenzhen Mindray Bio-Medical Electronics Co., Ltd., Shenzhen, China) with commercial reagent kits supplied by BioSino Bio-Technology & Science Inc. (Beijing, China). All assays were conducted according to the manufacturers’ instructions.

### 2.5 Widely targeted metabolomic profiling of herbal ingredients

Each of the five herbal ingredients included in the Shariga-5 formula (Table 2) was analyzed separately. One freeze-dried sample of each herb was ground using a mixer mill (MM 400, Retsch, Haan, Germany) for 1.5 min at 30 Hz. A 100-mg aliquot was extracted overnight at 4 °C with 1.0 mL of 70% aqueous methanol. After centrifugation at 10,000 × g for 10 min, the extract was cleaned using a CNWBOND Carbon-GCB solid-phase extraction cartridge (250 mg, 3 mL; ANPEL, Shanghai, China) and filtered through a 0.22-μm membrane filter before LC–MS/MS analysis.

Metabolite profiling was performed using a Shim-pack UFLC system (CBM-30A; Shimadzu, Kyoto, Japan) coupled to an API 4500 QTRAP mass spectrometer (AB Sciex, Framingham, MA, USA). Chromatographic separation was performed using a Waters ACQUITY UPLC HSS T3 C18 column (1.8 μm, 2.1 × 100 mm). The mobile phases were water containing 0.04% acetic acid (solvent A) and acetonitrile containing 0.04% acetic acid (solvent B). The gradient (A: B, v/v) was programmed as follows: 95:5 at 0 min, changed linearly to 5:95 at 11.0 min, held until 12.0 min, returned to 95:5 at 12.1 min, and held until 15.0 min. The flow rate was 0.40 mL/min, the column temperature was 40 °C, and the injection volume was 5 μL.

The mass spectrometer was equipped with an ESI Turbo Ion-Spray interface and operated in positive- and negative-ion modes under the control of Analyst software version 1.6.3. The source temperature was 550 °C, and ion source gas I, ion source gas II, and curtain gas were set at 55, 60, and 25 psi, respectively. Metabolite signals were acquired in multiple reaction monitoring mode, with the declustering potential and collision energy optimized for each transition.

Metabolites were putatively annotated by matching precursor ions, product ions, and MS/MS fragmentation patterns against the proprietary MetWare Database (MWDB), with reference to MassBank, KNAPSAcK, HMDB, MoTo DB, and METLIN. Relative metabolite abundances were determined from MRM signal intensities, and Class I and Class II categories were assigned according to the hierarchical annotations provided in the MWDB output.

### 2.6 Untargeted serum metabolomic analysis by UHPLC–MS/MS

Serum metabolites were extracted using a methanol-based protein precipitation procedure adapted from previously described methods (19, 20). Briefly, serum samples (100 μL) were transferred into microcentrifuge tubes and mixed with 400 μL of pre-chilled 80% methanol. After vortex mixing, the samples were incubated on ice for 5 min and centrifuged at 15,000 × g for 20 min at 4 °C. An aliquot of the resulting supernatant was diluted with LC–MS-grade water to a final methanol concentration of 53% and centrifuged again at 15,000 × g for 20 min at 4 °C. The final supernatant was collected for UHPLC–MS/MS analysis. A pooled quality-control (QC) sample was prepared by combining equal-volume aliquots from all experimental samples.

Metabolomic profiling was performed using a Dionex Ultimate 3,000 UHPLC system coupled to a Q Exactive series mass spectrometer (Thermo Fisher Scientific). Chromatographic separation was achieved using a Thermo Scientific Syncronis C18 column (2.1 × 100 mm, 1.7 μm) at a flow rate of 0.2 mL/min. Mobile phase A consisted of water containing 0.1% formic acid and 2 mmol/L ammonium formate, and mobile phase B consisted of acetonitrile. The gradient program was as follows: 95% A at 0–1 min; 95–40% A at 1–5 min; 40–0% A at 5–8 min; 0% A at 8–11 min; 0–40% A at 11–14 min; 40–95% A at 14–15 min; and 95% A at 15–18 min. Experimental samples were analyzed in a randomized order, and one pooled QC sample was injected after every six experimental samples to monitor analytical stability.

The mass spectrometer was equipped with an electrospray ionization source and operated in positive- and negative-ion polarity-switching mode. The spray voltage was set to 2.8 kV. The sheath and auxiliary gas flow rates were set to 35 and 10 arbitrary units, respectively, and the capillary temperature was maintained at 320 °C. Full-scan MS data were acquired at a resolution of 70,000 over an m/z range of 70–1,050. Data-dependent MS/MS acquisition was performed at a resolution of 17,500 using stepped normalized collision energies of 20, 40, and 60.

Raw data files were processed using TraceFinder version 3.2.0 (Thermo Fisher Scientific). Chromatographic features were aligned across samples using a retention-time tolerance of 0.2 min and a mass tolerance of 5 ppm, and relative metabolite abundances were determined from integrated peak areas. Metabolite features were putatively annotated by matching accurate precursor-ion masses and MS/MS fragmentation spectra against the mzCloud database and an in-house spectral library. According to the Metabolomics Standards Initiative criteria, these annotations were classified as level 2. Peak areas were normalized to the total peak area of each sample before subsequent statistical analysis.

### 2.7 Statistical analysis

Statistical analyses of serum mineral concentrations, antioxidant indices, biochemical parameters, and hormone levels were performed using IBM SPSS Statistics 27.0 (IBM Corp., Armonk, NY, USA). Data were tested for normality and homogeneity of variance before analysis. Differences between the MHMS and CON groups were analyzed using an independent-samples t-test. Results are presented as group means with the pooled SEM, and differences were considered significant at *p* < 0.05.

Metabolomic data were log-transformed and Pareto-scaled prior to multivariate analysis. Principal component analysis (PCA) and partial least squares-discriminant analysis (PLS-DA) were performed using SIMCA 14.1 (MKS Umetrics AB, Umeå, Sweden). Model performance was evaluated using R^2^Y and Q^2^ values, and a 200-permutation test was conducted to assess model overfitting. Differential metabolites between groups were identified using Welch’s t-test and PLS-DA-derived variable importance in projection (VIP) scores. Metabolites with VIP > 1.0, *p* < 0.05, and fold change (FC) ≥ 1.5 or FC ≤ 0.67 were considered differential metabolites. PCA and PLS-DA score plots, PLS-DA permutation test plots, and volcano plots of differential serum metabolites were visualized using the ggplot2 package in R. Metabolic pathway analysis (MetPA) was performed using MetaboAnalyst 6.0 based on the *Ovis aries* (sheep) pathway library in the KEGG database (21). Spearman correlation analysis was performed to evaluate the associations between differential metabolites and significantly altered serum mineral, antioxidant, biochemical, and hormone indicators. The correlation matrices were visualized using the pheatmap package in R.

## 3 Results

### 3.1 Secondary metabolite profiles of the five herbal ingredients

Widely targeted metabolomic profiling yielded 216, 265, 318, 325, and 303 metabolite annotations for Shariga, Saolong-Jimusi, Gaoletu-Baori, Arula, and Zhurula, respectively. In total, 1,427 herb-level metabolite annotation records were obtained across the five herbs. Phenolic acids were the most abundant category, accounting for 553 records (38.75%), followed by flavonoids (451 records, 31.60%), alkaloids (107 records, 7.50%), terpenoids (81 records, 5.68%), lignans and coumarins (80 records, 5.61%), tannins (55 records, 3.85%), and quinones (1 record, 0.07%) (Figure 2). Together, phenolic acids and flavonoids accounted for 70.35% of all herb-level annotation records.

**Figure 2:**
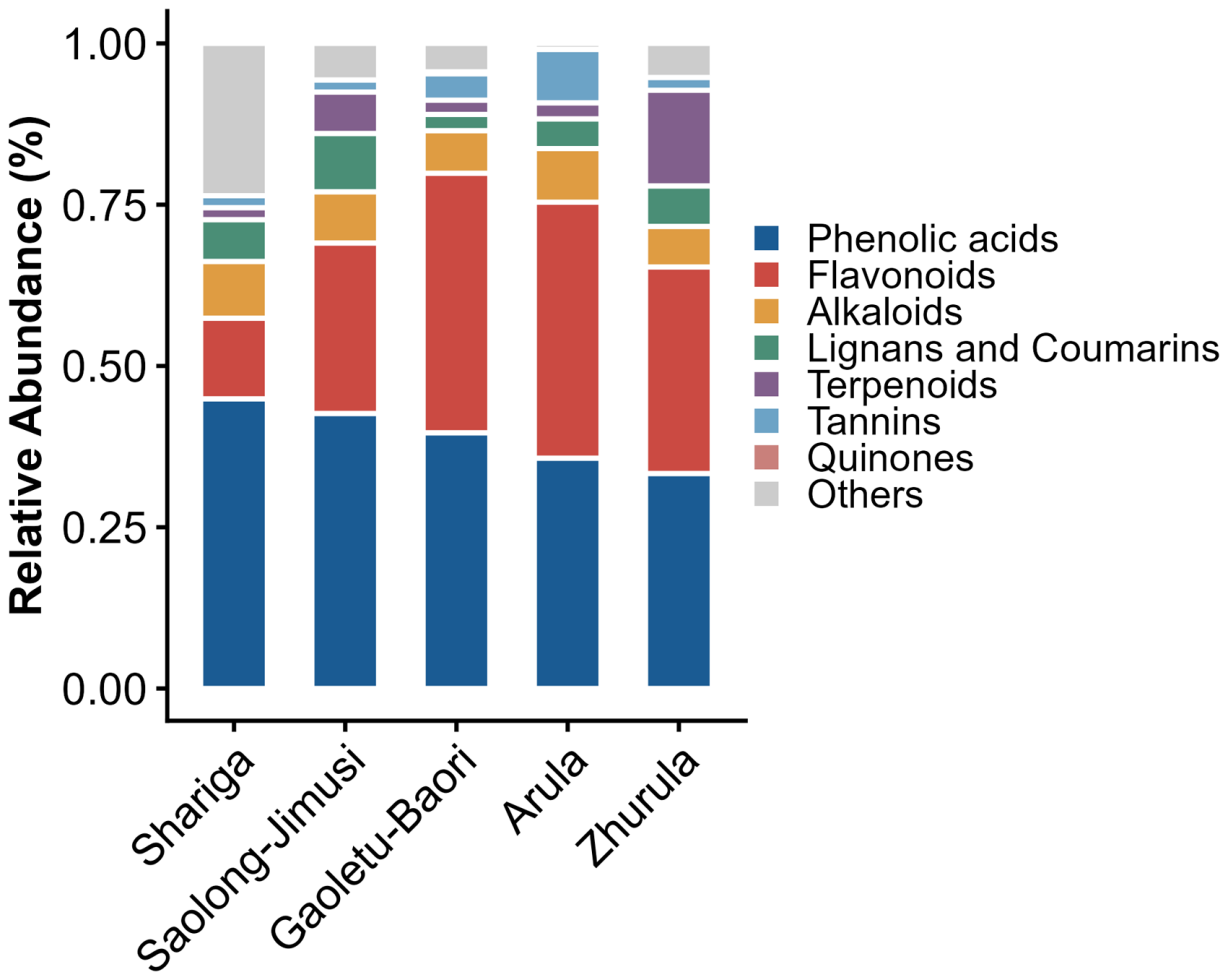
Class I distribution of herb-level metabolite annotation records obtained from the five medicinal herbs separately analyzed in the Shariga-5 formula. The numbers and percentages indicate the metabolite annotation records assigned to each Class I category.

### 3.2 Serum mineral concentrations

Serum mineral concentrations are presented in Table 4. Compared with the CON group, the MHMS group had higher serum concentrations of Cu (*p* = 0.012), Zn (*p* = 0.029), and Se (*p* = 0.016). No significant between-group differences were observed in serum Ca, P, Mg, Na, K, Fe, or Mn concentrations (*p* > 0.05).

**Table 4:** Effects of Mongolian herbal–mineral supplementation on serum mineral concentrations in sheep.

Items	Groups		SEM	*p*-value
	CON	MHMS		
Ca (mmol/L)	2.56	2.84	0.070	0.085
P (mmol/L)	2.45	2.25	0.144	0.505
Mg (mmol/L)	0.87	0.86	0.041	0.890
Na (mmol/L)	143.87	143.44	2.022	0.886
K (mmol/L)	5.33	5.18	0.264	0.788
Fe (μmol/L)	26.89	29.79	1.419	0.337
Cu (μmol/L)	4.86	9.55	0.569	0.012
Zn (μmol/L)	10.62	15.29	0.877	0.029
Mn (μmol/L)	0.33	0.33	0.009	0.800
Se (μmol/L)	0.43	0.59	0.027	0.016

CON, control; MHMS, Mongolian herbal–mineral supplement, Ca, calcium; P, phosphorus; Mg, magnesium; Na, sodium; K, potassium; Fe, iron; Cu, copper; Zn, zinc; Mn, manganese; Se, selenium; SEM, pooled standard error of the mean; *n* = 6/group.

### 3.3 Serum biochemical parameters

Serum biochemical parameters are presented in Table 5. Serum GLU concentration (*p* = 0.008) and AST activity (*p* = 0.004) were lower in the MHMS group than in the CON group. No significant between-group differences were observed in TP, ALB, UREA, CHO, TG, HDL-C, LDL-C, or ALT activity (*p* > 0.05).

**Table 5:** Effects of Mongolian herbal–mineral supplementation on serum biochemical parameters in sheep.

Items	Groups		SEM	*p-*value
	CON	MHMS		
GLU (mmol/L)	4.44	3.60	0.128	0.008
TP (g/L)	62.30	59.35	2.397	0.552
ALB (g/L)	26.83	25.75	3.049	0.863
UREA (mmol/L)	1.08	1.45	0.223	0.428
CHO (mmol/L)	1.74	1.94	0.075	0.211
TG (mmol/L)	0.08	0.12	0.009	0.050
HDL-C (mmol/L)	1.01	1.13	0.047	0.222
LDL-C (mmol/L)	0.29	0.34	0.017	0.164
ALT (U/L)	44.67	42.21	2.199	0.588
AST (U/L)	160.70	125.35	4.700	0.004

CON, control; MHMS, Mongolian herbal–mineral supplement, GLU, glucose; TP, total protein; ALB, albumin; UREA, urea; CHO, cholesterol; TG, triglycerides; HDL-C, high-density lipoprotein cholesterol; LDL-C, low-density lipoprotein cholesterol; ALT, alanine aminotransferase; AST, aspartate aminotransferase; SEM, pooled standard error of the mean; *n* = 6/group.

### 3.4 Serum antioxidant capacity

Serum antioxidant indices are presented in Table 6. Serum T-AOC was higher in the MHMS group than in the CON group (*p* = 0.017). Serum SOD, GSH-Px, and CAT activities and MDA concentration did not differ significantly between groups (*p* > 0.05).

**Table 6:** Effects of Mongolian herbal–mineral supplementation on serum antioxidant indices in sheep.

Items	Groups		SEM	*p*-value
	CON	MHMS		
T-AOC (U/mL)	0.46	0.51	0.009	0.017
SOD (U/mL)	7.96	7.69	0.266	0.627
GSH-Px (U/mL)	0.10	0.10	0.005	0.381
CAT (U/L)	121.11	105.83	11.776	0.531
MDA (nmol/mL)	0.25	0.56	0.074	0.063

CON, control; MHMS, Mongolian herbal–mineral supplement; T-AOC, total antioxidant capacity; SOD, superoxide dismutase; GSH-Px, glutathione peroxidase; CAT, catalase; MDA, malondialdehyde; SEM, pooled standard error of the mean; *n* = 6/group.

### 3.5 Serum hormone concentrations

Serum hormone concentrations are presented in Table 7. Compared with the CON group, the MHMS group had higher serum concentrations of T3, T4, IGF-1, and GH and a lower serum concentration of CORT (all *p* < 0.001).

**Table 7:** Effects of Mongolian herbal–mineral supplementation on serum hormone levels in sheep.

Items	Groups		SEM	*p*-value
	CON	MHMS		
T3 (pmol/L)	5.80	8.90	0.184	<0.001
T4 (nmol/L)	147.12	177.67	3.632	<0.001
IGF-1 (ng/mL)	73.90	98.71	2.422	<0.001
GH (μg/mL)	9.17	11.63	0.284	<0.001
CORT (pg/mL)	943.29	745.77	24.075	<0.001

CON, control; MHMS, Mongolian herbal–mineral supplement; T3, triiodothyronine; T4, thyroxine; IGF-1, insulin-like growth factor 1; GH, growth hormone; CORT, cortisol; SEM, pooled standard error of the mean; *n* = 6/group.

### 3.6 Serum metabolomic profiling and pathway analysis

A total of 1,062 metabolites were putatively annotated in serum samples from the CON and MHMS groups. PCA showed partial overlap between the two groups (Figure 3A), whereas the supervised PLS-DA score plot showed clearer group separation (Figure 3B). The two-component PLS-DA model yielded R^2^Y and Q^2^ values of 0.997 and 0.630, respectively, and permutation testing yielded a Q^2^ intercept of −0.059 (Figure 3C). Using the prespecified criteria of VIP > 1.0, nominal *p* < 0.05, and FC ≥ 1.5 or FC ≤ 0.67, 112 candidate differentially abundant metabolites were detected. Of these, 51 showed higher abundance and 61 showed lower abundance in the MHMS group than in the CON group (Figure 4).

**Figure 3:**
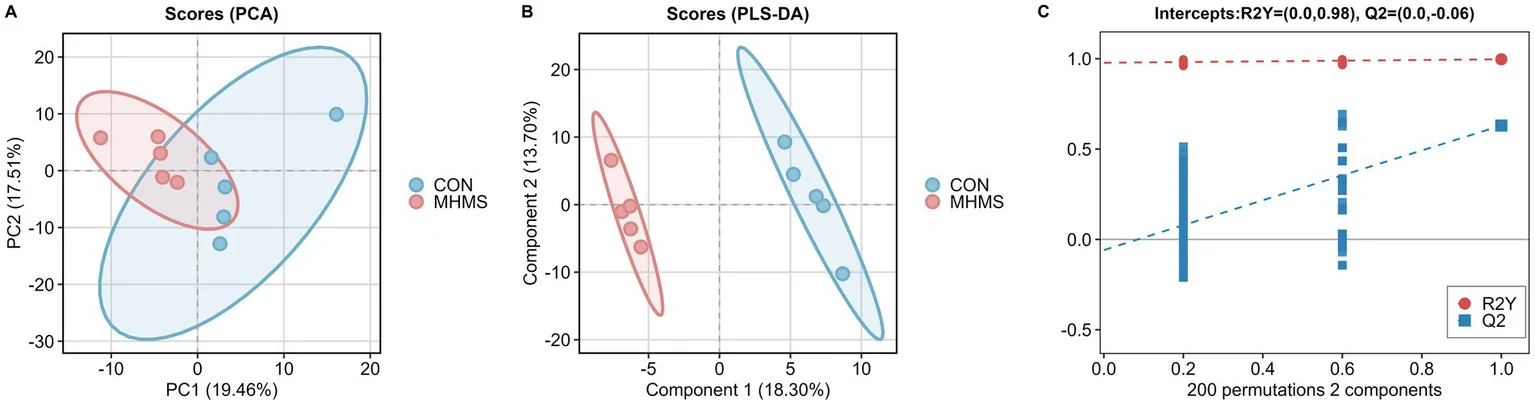
Multivariate analysis of serum metabolomic profiles in the control (CON) and Mongolian herbal–mineral supplement (MHMS) groups. **(A)** Principal component analysis (PCA) score plot. **(B)** Partial least squares–discriminant analysis (PLS–DA) score plot. **(C)** Results of the 200-permutation test used to evaluate potential overfitting of the PLS–DA model. Each point in the score plots represents an individual serum sample. R^2^Y, goodness of fit; Q^2^, predictive ability.

**Figure 4:**
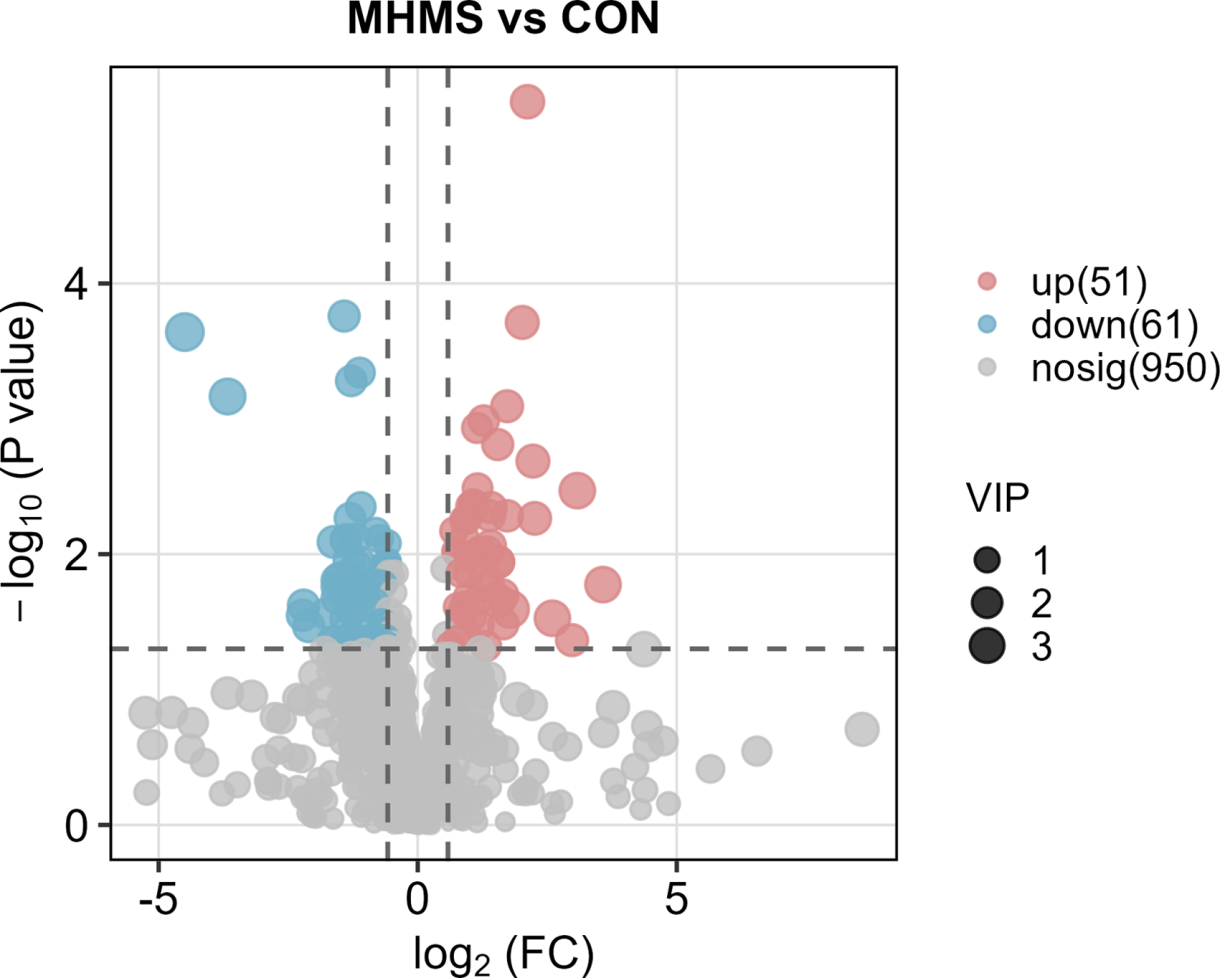
Volcano plot of candidate differential serum metabolites between the control (CON) and Mongolian herbal–mineral supplement (MHMS) groups. Candidate differential metabolites were selected using the criteria VIP > 1.0, *p* < 0.05, and fold change (FC) ≥ 1.5 or ≤ 0.67. Metabolites with increased and decreased relative abundance in the MHMS group are shown separately from metabolites that did not meet the selection criteria. VIP, variable importance in projection.

Pathway analysis ranked porphyrin metabolism, glutathione metabolism, glyoxylate and dicarboxylate metabolism, and arachidonic acid metabolism among the most strongly affected pathways (Figure 5). Porphyrin metabolism was nominally enriched (*p* = 0.036) but did not remain significant after FDR (FDR = 0.889) correction. Among the candidate metabolites mapped to this pathway, glycine abundance was higher (FC = 2.23), whereas porphobilinogen abundance was lower (FC = 0.39), in the MHMS group than in the CON group.

**Figure 5:**
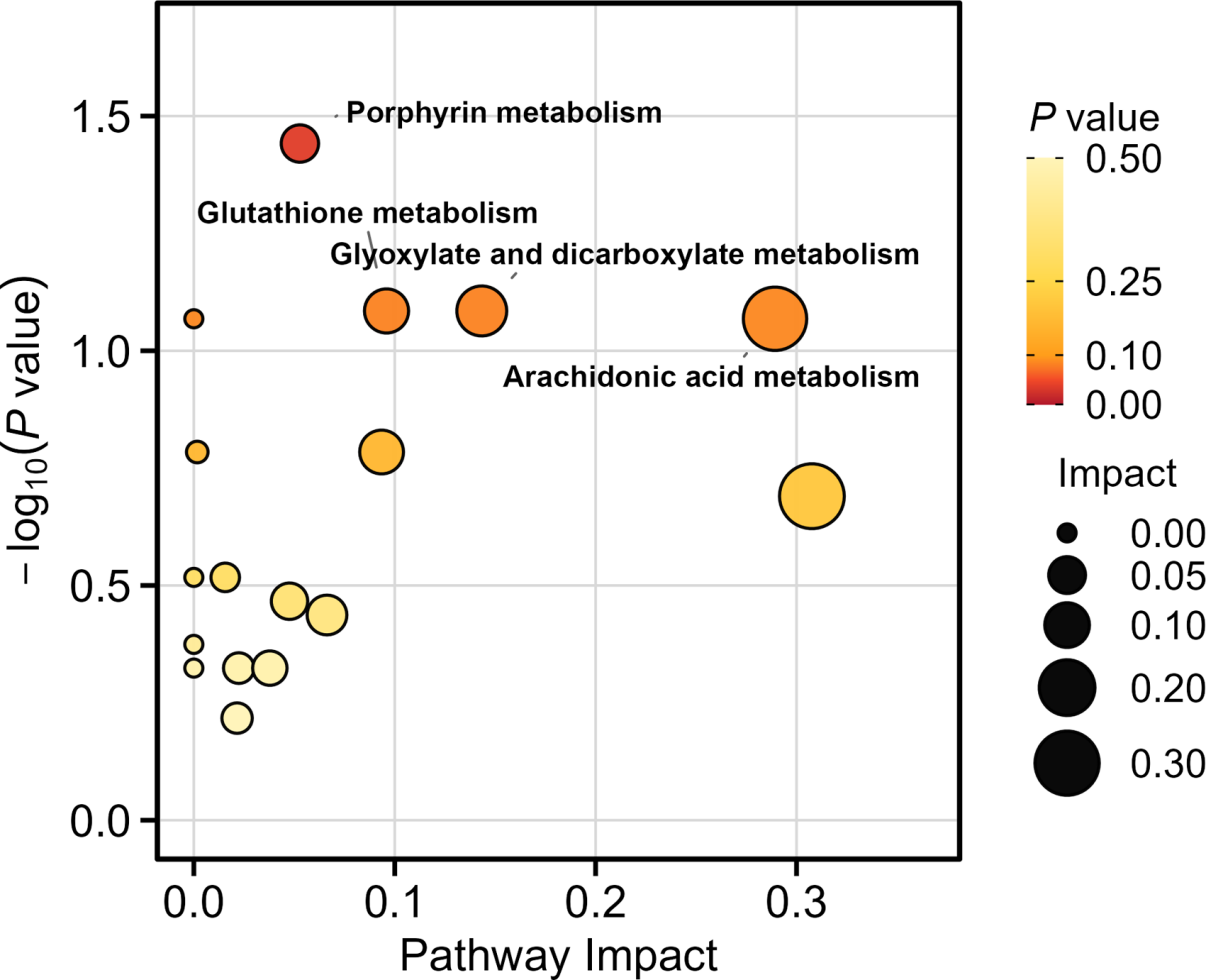
KEGG pathway analysis of candidate differential serum metabolites between the control (CON) and Mongolian herbal–mineral supplement (MHMS) groups. The position, size, and color of each point represent the pathway impact, number of matched metabolites, and pathway enrichment significance, respectively. Note: Differential metabolites were putatively annotated using mzCloud databases (presumed identification) without validation by authentic chemical standards.

Exploratory Spearman correlation analysis showed several associations between candidate metabolites and serum indicators (Figure 6). Porphobilinogen was positively correlated with serum AST activity. Glycine was positively correlated with T3, IGF-1, Cu, Zn, and Se.

**Figure 6:**
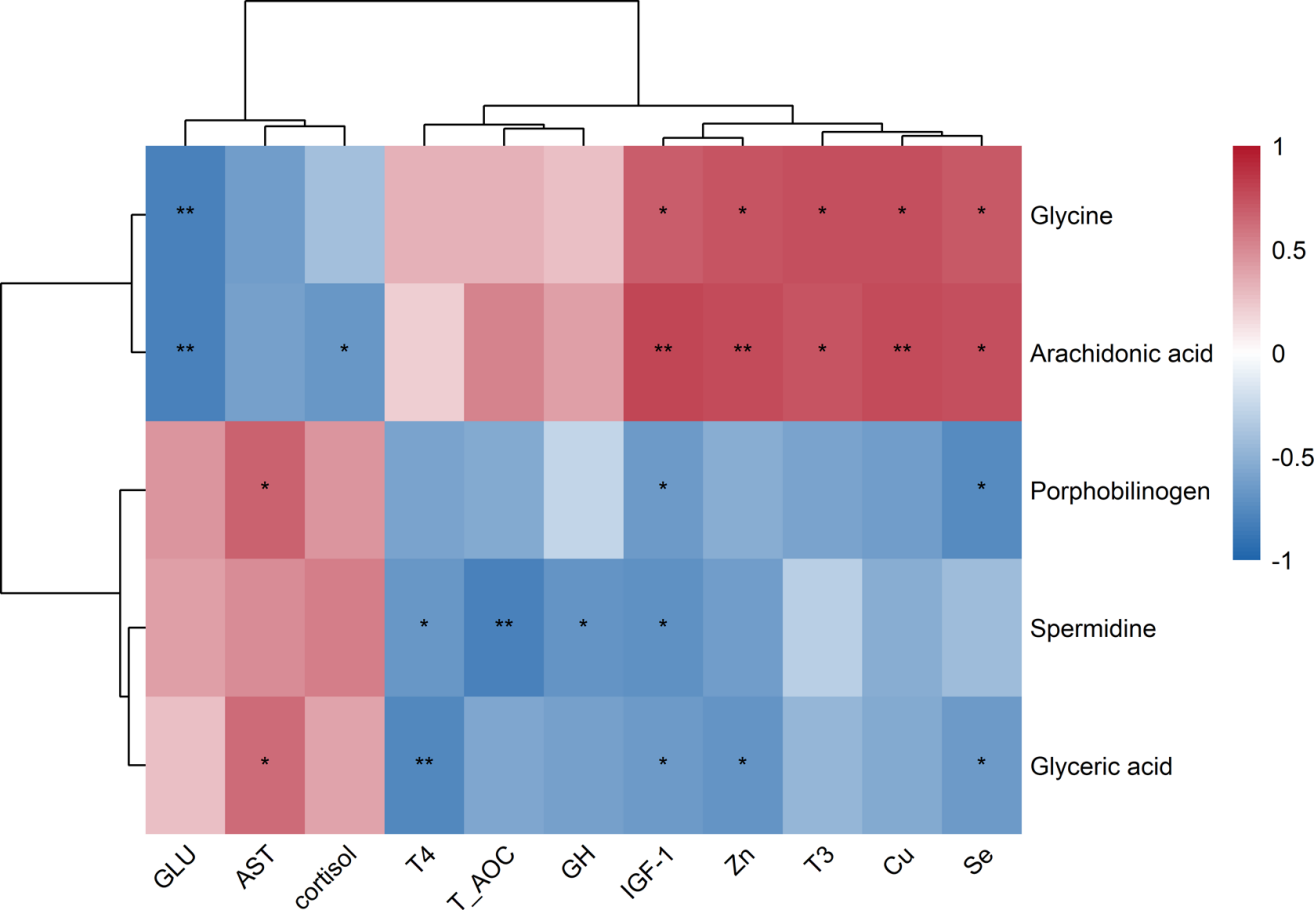
Spearman correlation heatmap showing associations between selected candidate differential serum metabolites and serum mineral, biochemical, antioxidant, and hormonal indicators that differed between the control (CON) and Mongolian herbal–mineral supplement (MHMS) groups. Colors represent the direction and magnitude of Spearman’s correlation coefficient.

## 4 Discussion

Mineral deficiency is a recognized correlate of pica in ruminants, but the phenotype may coexist with disturbances in redox, endocrine, and metabolic status (2, 4, 8, 9). The present findings support this more integrative view. MHMS not only directly supplied key trace elements, including Cu, Zn, and Se, but also appeared to enhance antioxidant status and alter endocrine profiles, with exploratory differences observed in serum metabolic profiles, including metabolites related to porphyrin metabolism. Together, these responses may help shift pica-affected sheep from a stress-associated metabolic state toward improved physiological homeostasis.

Unlike conventional mineral supplements, MHMS combines mineral elements with the Mongolian herbal formula Shariga-5. Secondary metabolite profiling of Shariga-5 showed that phenolic acids and flavonoids were the dominant phytochemical constituents of the five medicinal herbs. Phenolic acids and flavonoids possess a broad range of biological activities and can regulate redox homeostasis and immunometabolic status through free-radical scavenging, metal-ion chelation, modulation of enzymatic activity and regulation of inflammatory signaling (22, 23). Therefore, MHMS was designed as an integrated nutritional strategy intended to deliver both essential minerals and plant secondary metabolites. While essential trace elements directly underpin antioxidant enzyme activity and systemic metabolism, the concurrent provision of herbal bioactive compounds may offer complementary physiological support. This dual property may explain why the physiological responses observed in the present study were not restricted to changes in serum mineral concentrations, but extended broadly to antioxidant status, endocrine regulation and serum metabolic profiles.

The regulatory effect of MHMS on the physiological status of pica-affected sheep was first reflected in the improvement of key trace-element homeostasis. In the present study, MHMS significantly increased serum Cu, Zn and Se concentrations, whereas macroelements and other trace elements, including Ca, P, Mg, Na, K, Fe and Mn, were not significantly altered. This result suggests that MHMS primarily improved the status of key trace elements closely associated with redox defense, metalloenzyme activity, immune regulation and metabolic homeostasis. Copper is an essential component of various oxidoreductases and metalloenzymes and is involved in mitochondrial respiration and iron metabolism (24). Zinc acts as a structural and catalytic factor for numerous enzymes and is closely related to epithelial barrier function, immunity and antioxidant regulation (25, 26). Selenium is an essential component of selenoproteins, such as glutathione peroxidase, and directly participates in peroxide removal and maintenance of redox homeostasis (27). Thus, the increase in Cu, Zn and Se provides an important mineral basis for the subsequent effects of MHMS on antioxidant capacity, endocrine stress responses and serum metabolism. This selective response is also consistent with previous studies on mineral supplementation in ruminants. Jin et al. (28) investigated the effects of Ca, Zn, Cu, Co, Mn and Se deficiency and repletion on serum mineral status in grazing Mongolian sheep and found that mineral deficiency caused abnormalities in the serum mineral profile. Additional supplementation with Ca, Cu and Se significantly increased serum Ca, Cu and Se concentrations, whereas not all supplemented minerals increased in parallel. Meng et al. (29) reported that a Cu-deficient diet significantly reduced ruminal Cu content and apparent Cu absorption, while dietary Cu supplementation not only increased ruminal Cu content, but also significantly improved the apparent absorption of several minerals, including P, S, K, Ca, Mn, Fe, Cu and Zn. These results indicate that deficiency or supplementation of a single key trace element may influence the overall mineral utilization network in ruminants. Collectively, these studies suggest that serum mineral responses to dietary mineral supplementation are not determined solely by the amount supplied, but are also jointly affected by body mineral reserves, absorption efficiency, antagonistic interactions among minerals and homeostatic regulation (30, 31). Recent evidence in small ruminants highlights that such antagonistic imbalances can severely disrupt systemic trace-element homeostasis and trigger cascading physiological and oxidative disorders (32). In the context of the present study, the MHMS-induced increases in Cu, Zn and Se may reflect not only the direct supply of these elements from the supplement, but also a partial improvement in mineral absorption and utilization in pica-affected sheep.

MHMS increased serum T-AOC without detectable changes in SOD, GSH-Px, or CAT activity. This pattern indicates a change in composite antioxidant capacity rather than activation of the measured enzymes individually, because T-AOC integrates enzymatic and non-enzymatic antioxidant (33). Pica has been associated with increased oxidative stress and reduced antioxidant capacity (34). Under such conditions, supplementation with multiple trace minerals or plant-derived bioactive compounds may progressively restore redox homeostasis. Previous studies have shown that dietary supplementation with plantain and garlic leaves significantly increased serum total antioxidant capacity and the activities of key antioxidant enzymes, including glutathione peroxidase, superoxide dismutase and catalase, in sheep, suggesting that herbal supplementation can enhance free-radical scavenging capacity by activating the endogenous antioxidant enzyme system (35). Kholif et al. (36) reported that supplementation with a mixture of plant-derived feed additives significantly increased serum total antioxidant capacity in lactating dairy cows, indicating a positive regulatory effect of phytogenic components on systemic redox balance. The antioxidant activity of plant-derived feed additives is largely attributed to their richness in polyphenolic bioactive compounds, including phenolic acids, flavonoids, phenolic diterpenes and volatile oils (37). These compounds can scavenge excessive reactive oxygen species by donating hydrogen atoms or electrons, thereby interrupting oxidative chain reactions in tissues, and can also inhibit pro-oxidant activity through metal-ion chelation (38, 39). Therefore, the significant increase in serum T-AOC after MHMS supplementation may be related to the synergistic effects of plant-derived bioactive substances from Mongolian herbs and trace minerals, which enhanced the overall antioxidant defense without significantly altering the activity of any single antioxidant enzyme. Such enhancement in systemic antioxidant defenses is consistent with findings where dietary cecropin supplementation in goats similarly reinforced antioxidant capacity and suppressed lipid peroxidation (40). Among the minerals supplied by MHMS, Se, Fe and Zn are important components of the antioxidant enzyme system and play essential roles in animal growth and health through their involvement in antioxidant defense (41, 42). Previous studies have shown that supplementation with organic trace minerals under heat-stress conditions significantly improved the total antioxidant status of Holstein calves (43). Deficiencies in serum Fe, Cu and Se, together with reduced antioxidant capacity, may contribute to the occurrence of pica symptoms in ruminants, whereas preventive trace-mineral supplementation can help strengthen antioxidant defense (4). Notably, serum MDA exhibited an upward trend from 0.25 to 0.56 nmol/mL (*p* = 0.063) despite enhanced T-AOC. In a modest cohort, this twofold increase warrants attention. While higher T-AOC likely reflected non-enzymatic radical scavenging by herbal polyphenols, elevated MDA may stem from transient Cu-induced Fenton-like pro-oxidant pressure (44). Additionally, variable voluntary intake and limited statistical power (*n* = 6) likely contributed to this divergence.

Serum biochemical indices are important indicators for evaluating the overall metabolic status and liver function of ruminants (45). MHMS significantly decreased serum GLU and AST levels, whereas indices related to protein metabolism (TP, ALB and UREA), lipid metabolism (CHO, TG, HDL-C and LDL-C) and ALT activity were not significantly affected. The normal physiological reference range for blood glucose in sheep is approximately 2.78–4.44 mmol/L (46). In the CON group, serum glucose was 4.44 mmol/L, which was at the upper limit of the normal reference range, whereas GLU in the MHMS group decreased significantly to 3.60 mmol/L. This result indicates that MHMS did not induce an abnormal reduction in blood glucose, but rather shifted glucose concentration toward a more stable physiological range. In ruminants, dietary carbohydrates are extensively degraded by rumen microorganisms and produce little directly absorbable glucose. Therefore, the glucose required by the body is mainly synthesized through hepatic gluconeogenesis, and circulating glucose concentration reflects the efficiency of energy production and utilization (47, 48). Pica-affected animals are often accompanied by mineral imbalance and disordered metabolic regulation (6). Trace-mineral deficiency may impair the function of multiple key enzymes involved in energy and carbohydrate metabolism, thereby reducing energy utilization efficiency and disturbing glucose homeostasis (49). Plant-derived bioactive compounds and trace minerals have been shown experimentally to improve insulin sensitivity and glucose uptake and to influence glucose-related metabolic pathways, which may contribute to improved metabolic balance (50). In the present study, MHMS maintained blood glucose at a lower level within the normal physiological range, which may alleviate the metabolic burden under long-term hyperglycemic conditions. AST is a sensitive indicator of liver metabolic burden and tissue injury in sheep (51). According to previous reports, the normal reference range for serum ALT in sheep is <33 U/L (52), whereas that for AST is 64–158 U/L (53). In the present study, ALT levels in both the CON and MHMS groups were above the normal range. Although ALT was lower in the MHMS group than in the CON group, the difference was not significant. By contrast, the AST concentration in the CON group was 160.70 U/L, exceeding the normal range, whereas MHMS supplementation significantly reduced AST and restored it to the normal physiological range. Previous studies have reported that serum ALT and AST are significantly elevated in calves with pica compared with healthy calves, and further suggested that these increases may be associated with altered hepatic metabolism caused by Cu deficiency (54). In addition, long-term deficiency of essential minerals, such as Ca, Cu, Co, Mn and Se, in sheep can impair liver, myocardial and pancreatic function and lead to abnormal serum biochemical indicators related to liver function. Supplementation with Ca, Cu and Se can significantly increase their serum concentrations, whereas supplementation with Zn, Co, Mn and Se helps reduce liver-related biochemical indices and alleviate liver injury (28). Given that elevated serum AST is not exclusively liver-specific but also associated with muscle damage (51, 55), this mineral replenishment may possibly also improve skeletal muscle metabolism. These findings provide strong support for the present observation that MHMS significantly increased Cu, Zn and Se concentrations while reducing AST levels, suggesting that improvement in key trace-element status may participate in alleviating tissue metabolic stress in pica-affected sheep.

The bioactive herbal components in MHMS may also have contributed to the decrease in AST. Previous studies have shown that dietary inclusion of an equal-ratio mixture of *Woodfordia fruticosa*, *Solanum nigrum* and *Trigonella foenum-graecum* at 3% of dry matter intake significantly reduced serum AST levels in goats, whereas ALT remained unchanged (56). This pattern is similar to the present finding that AST decreased significantly, while ALT did not change significantly. Together with the significant increase in T-AOC observed in this study, these results suggest that MHMS may enhance antioxidant buffering capacity through the combined effects of key trace-element compensation and plant-derived bioactive compounds, thereby reducing oxidative stress-associated tissue metabolic pressure. This mechanism may partly explain the decrease in AST activity.

In the present study, MHMS significantly increased serum T3, T4, IGF-1 and GH concentrations and significantly decreased CORT concentration, suggesting that MHMS may improve endocrine–metabolic adaptation in pica-affected sheep. Thyroid hormones T3 and T4 are important endocrine regulators of energy metabolism and metabolic rate (57). The increased T3 and T4 concentrations observed in this study indicate that MHMS may enhance metabolic activity and energy turnover in pica-affected sheep. The simultaneous increases in GH and IGF-1 suggest activation of anabolic endocrine signaling, which may reflect an improvement in tissue metabolism and growth regulation (58). Previous studies have shown that dietary curcumin supplementation significantly increased T3 and T4 levels and reduced cortisol levels in Zaraibi goats (59). Singh et al. (60) similarly reported that supplementation with *Curcuma longa* and *Allium sativum*, or *Curcuma longa* and *Ocimum sanctum*, increased blood T3 and T4 concentrations and decreased cortisol levels in Black Bengal goats. These findings are broadly consistent with the regulatory effects of MHMS on T3, T4 and CORT observed in the present study, suggesting that plant secondary metabolites derived from Mongolian herbs may be involved in thyroid hormone homeostasis and endocrine regulation of metabolism. Cortisol is the major stress hormone, and its reduction indicates alleviation of chronic stress (61, 62). The hypothalamic–pituitary–adrenal axis (HPA) is the central endocrine pathway mediating the stress response. Chronic stress can trigger sustained activation of this axis and promote cortisol release (63). Glucocorticoids derived from HPA axis activation can further induce responses in the cellular redox system (64). Therefore, nutritional imbalance and oxidative stress associated with pica may increase HPA axis burden and lead to elevated cortisol levels. In the present study, MHMS contained Se, Cu, Zn and vitamin E, all of which have direct or indirect antioxidant functions (65), providing a possible basis for the cortisol-lowering effect of MHMS. Previous studies have shown that vitamin E and Se supplementation reduced serum cortisol levels and improved total antioxidant status in dairy cows subjected to transportation stress (66). Organic mineral supplementation has also been reported to effectively reduce cortisol concentrations in heat-stressed Holstein bull calves, indicating a positive role in regulating stress-related hormone secretion (43). Thus, MHMS may reduce oxidative stress-related endocrine stress responses by improving mineral supply and antioxidant defense, thereby contributing to lower CORT concentrations.

Untargeted serum metabolomic analysis identified porphyrin metabolism as a nominally enriched pathway between the CON and MHMS groups; however, this enrichment did not remain significant after FDR correction and should therefore be interpreted as exploratory. Porphyrin metabolism is a central pathway for heme synthesis and heme homeostasis. As an iron–porphyrin prosthetic group, heme is essential for the function of hemoglobin, myoglobin, cytochromes and various oxidoreductases, and is involved in oxygen transport, mitochondrial electron transfer, energy metabolism and maintenance of redox homeostasis (67). In the present study, porphobilinogen abundance was lower, and glycine abundance was higher in the porphyrin metabolism pathway in the MHMS group. Spearman correlation analysis showed that porphobilinogen was positively correlated with serum AST, whereas glycine was positively correlated with serum T3, IGF-1, Cu, Zn and Se. Glycine is a key substrate for the early steps of heme synthesis. The initial rate-limiting reaction of heme biosynthesis is catalyzed by ALA synthase, in which glycine condenses with succinyl-CoA to produce δ-aminolevulinic acid (ALA). Subsequently, two ALA molecules are condensed by ALA dehydratase to form porphobilinogen (PBG), which then participates in downstream tetrapyrrole structure formation and heme synthesis (68). Previous studies have shown that when the early metabolic flux of heme synthesis is impaired, particularly under insufficient porphobilinogen deaminase (PBGD) activity, PBG may accumulate abnormally and is often accompanied by increased ALA. Excessive PBG can also inhibit δ-aminolevulinic acid dehydratase (ALAD) activity, further disrupting the metabolic balance between ALA and PBG (69). Therefore, the downregulation of PBG in the MHMS group may be associated with reduced abnormal accumulation of early porphyrin precursors under pica recovery. The positive correlation between PBG and serum AST suggests that higher PBG levels may be associated with increased tissue metabolic pressure or hepatic metabolic burden. Because pica is generally associated with mineral deficiency and impaired antioxidant defense capacity (4, 34, 70), the higher glycine abundance may also be relevant to antioxidant metabolism. Glycine is an important amino acid precursor required for glutathione synthesis (71). Previous studies have reported that under high oxidative stress in dairy cows, glutathione (GSH) synthesis processes related to glycine, glutamate and cysteine metabolism in the hindgut were weakened, accompanied by reduced plasma GSH and GSH-Px levels (72). Randomized clinical trials have also shown that supplementation with GlyNAC, composed of glycine and N-acetylcysteine, can improve glutathione deficiency and reduce oxidative stress and oxidative damage (73). The positive correlations of glycine with Cu, Zn and Se further suggest that glycine-related antioxidant substrate supply may cooperate with key trace-element-dependent antioxidant defense in the changes consistent with greater total antioxidant capacity. Importantly, these metabolomic observations and correlation patterns reflect phenotypic associations rather than definitive causal drivers of the observed physiological improvements, and establishing the direct regulatory role of porphyrin pathway intermediates requires further mechanistic verification. Collectively, the upregulation of glycine and downregulation of porphobilinogen after MHMS supplementation suggest potential associations between the Mongolian herbal–mineral lick block and the modulation of oxygen transport, energy metabolism and antioxidant defense in pica-affected sheep. These associations might be linked to heme precursor metabolism, the reduction in abnormal accumulation of metabolic intermediates, and the provision of glutathione-related antioxidant substrates, though further targeted validation is warranted. This interpretation is consistent with a recent multi-omics study in goats, in which dietary probiotic supplementation was associated with coordinated changes in the gut microbiota, hepatic antioxidant signaling, and arachidonic acid metabolism, together with reduced intestinal and hepatic inflammation and oxidative stress (74). These findings provide exploratory, hypothesis-generating insights into the metabolic correlates associated with MHMS supplementation in pica-affected sheep.

In ruminants, orally administered supplements first interact with the rumen environment before post-ruminal absorption. Dietary trace mineral variations have been shown to alter ruminal microbial populations and tissue mineral distribution in goats (75), while supplementation with plant-derived feed ingredients can modify rumen bacterial composition, fermentation, and metabolite profiles in sheep (76). Because rumen fluid and microbiota were not examined in the present study, the observed serum changes in glycine and porphobilinogen were likely linked to upstream ruminal responses to MHMS, though direct validation remains to be established.

Several limitations of the present study should be acknowledged. First, because MHMS was provided via continuous ad libitum access to lick blocks, individual intake was not recorded; consequently, the exact administered dose and potential exposure–response relationships cannot be determined. Second, comparing composite MHMS against an unsupplemented control precludes distinguishing whether improvements stem independently from minerals, herbal bioactive compounds, or their synergistic interactions. Third, as East Friesian sheep exhibit substantial individual genetic variation in metabolic efficiency and endocrine regulation, the lack of pedigree records to establish full-sib or half-sib cohorts represents a potential source of genetic noise within this modest sample size. Finally, although untargeted metabolomics revealed coordinated alterations in the porphyrin pathway, this enrichment did not withstand FDR correction, and crucially, the lack of validation by authentic standards for these presumed metabolites represents a major limitation of this study. Future multi-arm factorial trials incorporating pedigree-controlled cohorts, individual intake monitoring, and targeted validation against authentic standards are warranted to corroborate these findings.

## 5 Conclusion

In summary, free-choice supplementation with MHMS increased serum Cu, Zn and Se concentrations, enhanced serum T-AOC, elevated serum T3, T4, IGF-1 and GH concentrations, and decreased serum CORT concentration. Serum metabolomic profiling further indicated changes in metabolic pathways, particularly porphyrin metabolism, characterized by increased glycine abundance and decreased porphobilinogen abundance (Figure 7). Collectively, these findings clarify that MHMS primarily acts to alleviate oxidative stress by supplementing essential trace element cofactors and regulating the metabolic flow of the glycine and heme synthesis pathways, thereby supporting endocrine-metabolic adaptation in sheep with pica. Nonetheless, although oral supplementation significantly improved serum mineral levels and metabolic indicators, it did not completely reverse all abnormal indicators, suggesting that the full physiological recovery from pica may require a longer period of tissue repair and systemic remodeling.

**Figure 7:**
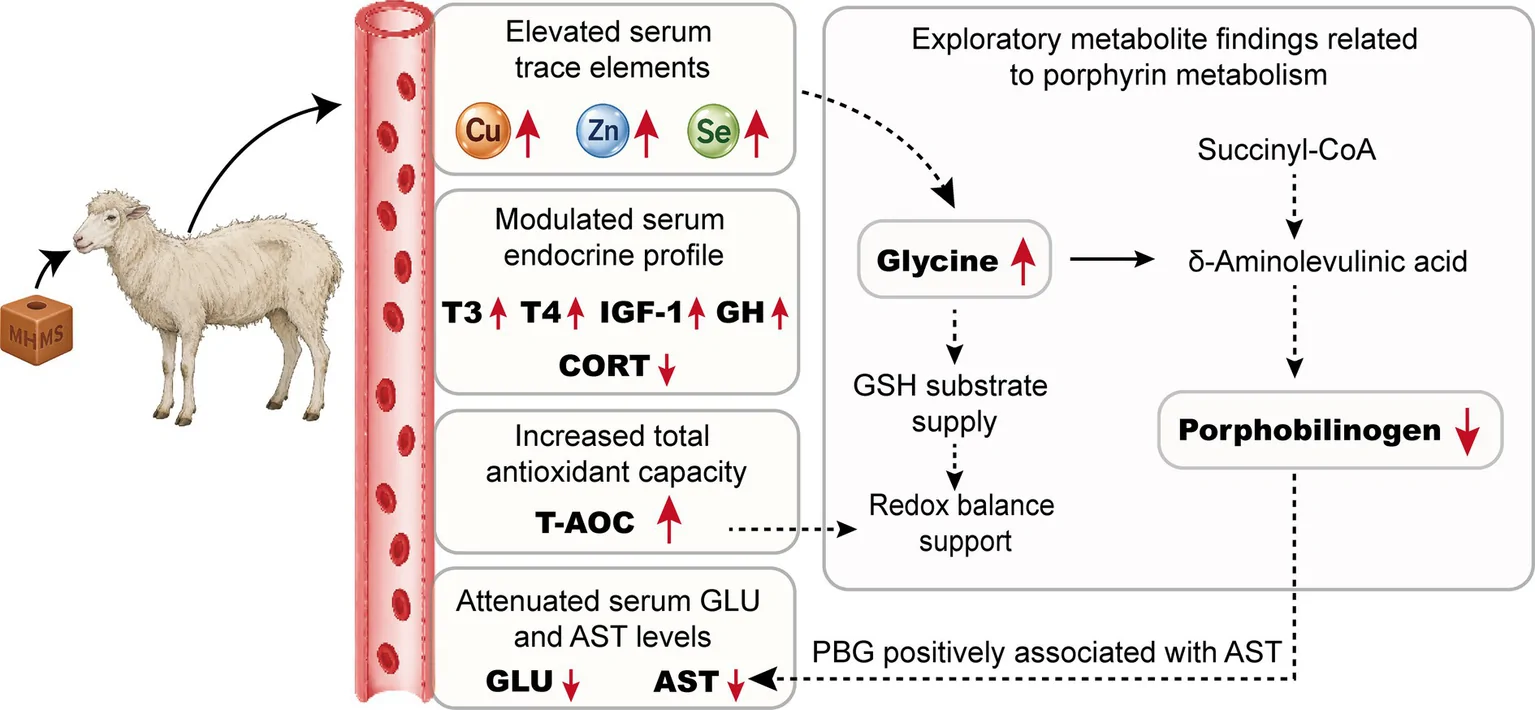
Conceptual summary of the observed serum differences and exploratory metabolite associations following free-choice supplementation with a Mongolian herbal–mineral supplement (MHMS) in sheep with pica. Upward and downward red arrows indicate higher and lower values, respectively, in the MHMS group relative to the CON group. Solid arrows denote established biochemical steps, whereas dashed arrows represent putative regulatory pathways or exploratory statistical associations. AST = aspartate aminotransferase; CoA = coenzyme A; CORT = cortisol; Cu = copper; GH = growth hormone; GLU = glucose; GSH = glutathione; IGF-1 = insulin-like growth factor 1; MHMS = Mongolian herbal–mineral supplement; PBG = porphobilinogen; Se = selenium; T3 = triiodothyronine; T4 = thyroxine; T-AOC = total antioxidant capacity; Zn = zinc.

## Data Availability

The raw data supporting the conclusions of this article will be made available by the authors, without undue reservation.
